# Increase in national intravenous thrombolysis rates for ischaemic stroke between 2005 and 2012: is bigger better?

**DOI:** 10.1186/s12883-016-0574-7

**Published:** 2016-04-21

**Authors:** S. Scherf, M. Limburg, R. Wimmers, I. Middelkoop, H. Lingsma

**Affiliations:** Department of Neurology, Canisius Wilhelmina ziekenhuis, Nijmegen, Netherlands; Department of Neurology, Flevoziekenhuis, Almere, Netherlands; Stroke Knowledge Network Netherlands, Maastricht, Netherlands; Dutch Heart Foundation, The Hague, Netherlands; Department of Public Health, Erasmus MC, Rotterdam, Netherlands

**Keywords:** Ischaemic stroke, Thrombolysis, Registries, Volume-outcome associations, Quality of care

## Abstract

**Background:**

Intravenous thrombolytic therapy after ischaemic stroke significantly reduces mortality and morbidity. Actual thrombolysis rates are disappointingly low in many western countries. It has been suggested that higher patient volume is related to shorter door-to-needle-time (DNT) and increased thrombolysis rates. We address a twofold research question: a) What are trends in national thrombolysis rates and door-to-needle times in the Netherlands between 2005–2012? and b) Is there a relationship between stroke patient volume per hospital, thrombolysis rates and DNT?

**Methods:**

We used data from the Stroke Knowledge Network Netherlands dataset. Information on volume, intravenous thrombolysis rates, and admission characteristics per hospital is acquired through yearly surveys, in up to 65 hospitals between January 2005 and December 2012. We used linear regression to determine a possible relationship between hospital stroke admission volume, hospital thrombolysis rates and mean hospital DNT, adjusted for patient characteristics.

**Results:**

Information on 121.887 stroke admissions was available, ranging from 7.393 admissions in 2005 to 24.067 admissions in 2012. Mean national thrombolysis rate increased from 6.4 % in 2005 to 14.6 % in 2012. Patient characteristics (mean age, gender, type of stroke) remained stable. Mean DNT decreased from 72.7 min in 2005 to 41.4 min in 2012. Volume of stroke admissions was not an independent predictor for mean thrombolysis rate nor for mean DNT.

**Conclusion:**

Intravenous thrombolysis rates in the Netherlands more than doubled between 2005 and 2012, in parallel with a large decline in mean DNT. We found no convincing evidence for a relationship between stroke patient volume per hospital and thrombolysis rate or DNT.

## Background

Intravenous thrombolytic therapy (IVT) within 4,5 h after onset of ischaemic stroke results in a significant reduction in mortality and morbidity [1, 2]. If treatment delay is avoided, up to 24 % of stroke patients may be eligible for thrombolysis [3]. National data are mostly lacking, but studies have reported disappointingly low thrombolysis rates in many countries, e.g., United Kingdom (1,4 % in 2008) [4], Germany (8.4 in 2009) [5], Sweden (6,7 between 2007 and 2010) [6] and the USA (2,4 % in 2006) [7]. Regional differences in IVT have been described with much higher thrombolysis rates in some regions. E.g. in 15,7 % in South London in [8] and up to 35 % in the German state of Hesse in 2007–2008 [9]. In most countries, including the Netherlands, actual thrombolysis numbers and trends in national rates are unknown.

In various fields, especially in surgical specialties, a relationship between volume of treated patients and outcome has been demonstrated [10, 11]. Directed by the Leapfrog group, this finding has led to volume-based referral strategies and the introduction of volume thresholds in complex surgical interventions [12]. Some studies have suggested a positive relationship between volume of stroke patients per center and outcome [13–15]. Other studies did not confirm this relationship [16, 17] or questioned the actual meaning of the correlation, as a volume-outcome effect does not identify the underlying mechanisms through which higher case volume can translate into better outcomes [18, 19]. One possible mechanism through which high volumes may result in better outcomes is the improvement of logistic processes, which may reduce treatment delay and increase thrombolysis rates.

As the debate for stroke care is on-going, we aimed to assess the relationship between stroke patient volume, thrombolysis rates and DNT in a national stroke registry. We address a twofold research question: a) What are trends in national thrombolysis rates and door-to-needle times in the Netherlands between 2005–2012? and b) Is there a relationship between stroke admission volume per hospital, thrombolysis rates and DNT?

## Methods

### Study population

We used data on stroke admissions from the Knowledge Network Stroke Netherlands (KNSN) dataset. The scientific advisory board of the KNSN registration permitted of the analysis. Anonymised data sets are available for scientific research and can be obtained after approval by the scientific advisory board. KNSN foundation was set up in 2006 with the aim of implementing the Helsingborg declaration for stroke care of 2006 [20]. One of the targets of this European stroke consensus conference was the organization of national registries to be used for improvement of stroke care. In the Netherlands KNSN yearly sends out surveys to all participating hospitals inquiring about numbers of stroke patients, patient characteristics, average thrombolysis rates and average DNT [21]. Average thrombolysis rates and DNT were based on individual stroke registries of participating hospitals. In the first survey data on the year 2005 were asked retrospectively. From 2005 to 2008 data was collected at stroke service level which sometimes comprised multiple hospitals. Information of 11 (in 2005 and 2006) and 18 (in 2007) hospitals was aggregated. From 2008 registration continued on individual hospital level. Reported numbers are yearly averages at hospital level. All participating hospitals receive a yearly report containing their benchmark data in comparison with national averages. Participating hospitals have a local cooperation with rehabilitation facilities, nursing care and home nursing care, more or less consolidated in a formal local stroke service. Hospitals included were urban and rural as well as academic and non-academic hospitals. Since KNSN started in 2006, there has been a steady increase in the number of participating hospitals.

### Definitions

The available data were on hospital level and included total number of stroke admissions, number of admissions for ischaemic stroke, mean age, and gender. Information on thrombolysis included the number of ischaemic stroke admissions with IVT and mean DNT. Hospitals were categorized as academic hospitals, non-academic referral centers (non-academic hospitals with local function as referral center) or community hospitals (hospital with purely local function).

### Statistical analysis

We calculated the total number of stroke admissions and admissions for ischaemic stroke per year by summation of these numbers for all participating hospitals. Per hospital we calculated ischaemic stroke rate as number of admissions with ischaemic stroke divided by the total number of stroke admissions, and thrombolysis rate as number of admissions with IVT divided by total number of admissions for ischaemic stroke. Mean age and percentage males were calculated by using mean age and percentage males per hospital weighed by total number of stroke admissions per hospital. Overall thrombolysis rate was calculated using the thrombolysis rate per hospital weighed by the number of admissions with ischaemic stroke. Because of a stabilisation of the thrombolysis rate between 2010 and 2012 we performed a sensitivity analysis by excluding the seven hospitals who entered the registry in 2011. Mean DNT was calculated by average DNT per hospital weighed by number of admissions with IVT. To exclude the possibility that trends in IVT were mainly caused by a change in participating hospitals after the year of 2008 we performed a sensitivity analysis in which we excluded all hospitals who participated in 2008 in the year of 2011 and compared mean DNTs. We calculated means and interquartile ranges (IQR) at hospital level for volume, mean age, percentage male, thrombolysis rates and DNT over the years 2005 until 2012.

For analysis of the relationship between volume, thrombolysis rates and mean DNT we used data from 2012 since it included the highest number of participating hospitals and patients. Correlations between volume of stroke admissions, mean age, gender and type of hospital and thrombolysis rate and mean DNT were described with Pearson correlation coefficients (r). The differences between academic, non-academic referral centers and community hospitals in thrombolysis rates were tested using chi square statistics. The differences in type of hospital and DNT were tested with one-way ANOVA test. With multivariable linear regression we assessed whether volume of stroke admissions, mean age, gender and type of hospital were independent predictors of thrombolysis rate and mean DNT. Because of a low number of academic hospitals we combined academic and non-academic referral hospitals. All analyses were conducted using SPSS Version 20. Graphs were created in R (R version 2.12.0, The R Foundation for Statistical Computing).

## Results

### Study population

Participation in the KNSN registry increased from 19 hospitals in 2005 to 65 in 2012, i.e., the majority of all 86 Dutch hospitals. Of these 65 hospitals there were 4 academic hospitals, 23 non-academic referral hospitals and 38 community hospitals. In total we included 121.887 stroke admissions, ranging from 7.393 in 2005 to 24.067 in 2012. Of all admission 103,809 (85.2 %) were ischaemic strokes of which 12,258 (11.8 %) were thrombolysed. Table 1 presents the main characteristics of all included stroke admissions. Based on the data for the year 2012, there was limited variation in mean age and gender between the hospitals (IQR mean age = 71.0–73.0, IQR percentage males = 48.4–52.6). Volume varied substantially (IQR = 234–463 admissions per year). Also thrombolysis rate and DNT varied largely between the hospitals. (IQR thrombolysis rate = 12.1–17.3, IQR DNT = 38.0–47.0).

**Table 1:** Characteristics of included stroke admissions

Year	2005	2006	2007	2008	2009	2010	2011	2012
Hospitals (*N*)	19^a^	19^a^	28	42	44	55	63	65
Stroke admissions (*N*)	7393	7545	11,323	17,698	14,074	17,993	21,794	24,067
Ischemic strokes (*N*)	6117	6278	9585	14,780	11,916	15,473	18,889	20,771
Rate of ischemic stroke % (SD)	82.7(5.4)	83.2 (7.9)	84.7(3.4)	83.5(4.7)	84.7(5.4)	86.0(3.8)	86.7(3.8)	86.3(4.0)
Mean age (SD)	71.6(1.6)	71.5(1.5)	72.1(1.4)	72.4(2.0)	71.3(1.9)	71.7(1.7)	72.0(1.7)	71.6 (2.1)
Gender male % (SD)	49.7(2.9)	50.5(3.6)	50.6(2.2)	50.1(3.7)	51.1(4.9)	49.8(3.8)	50.5(4.6)	50.9(3.3)

^**a**^From 2005 to 2008 data was collected at stroke service level which sometimes comprised multiple hospitals. Information of 11 (in 2005 and 2006) and 18 (in 2007) hospitals was aggregated

### Trends in IVT rates and DNT

**Fig. 1:**
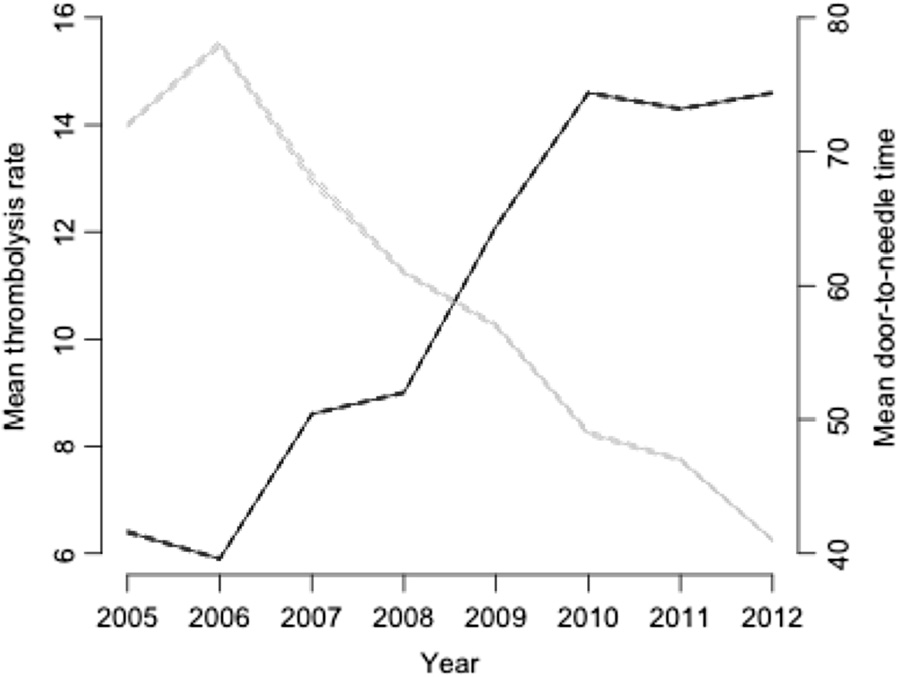
The black line represents the mean thrombolysis rate per year. The grey line represents the mean door-to-needle time per year

National thrombolysis rate increased from 6.4 % [95 % confidence interval (CI), 6.2–6.5 %] in 2005 to 14.6 % [95 % CI 14.5–14.6 %] in 2012 (Fig. 1). In 2011 there was a stabilisation of the thrombolysis rate. Mean DNT decreased from 72.2 min [95 % CI 71.4–74.0] in 2005 to 41.4 min [95 % CI 41.1–41.6] in 2012.

### Relationships between volume of stroke admissions, IVT rate and DNT

**Fig. 2:**
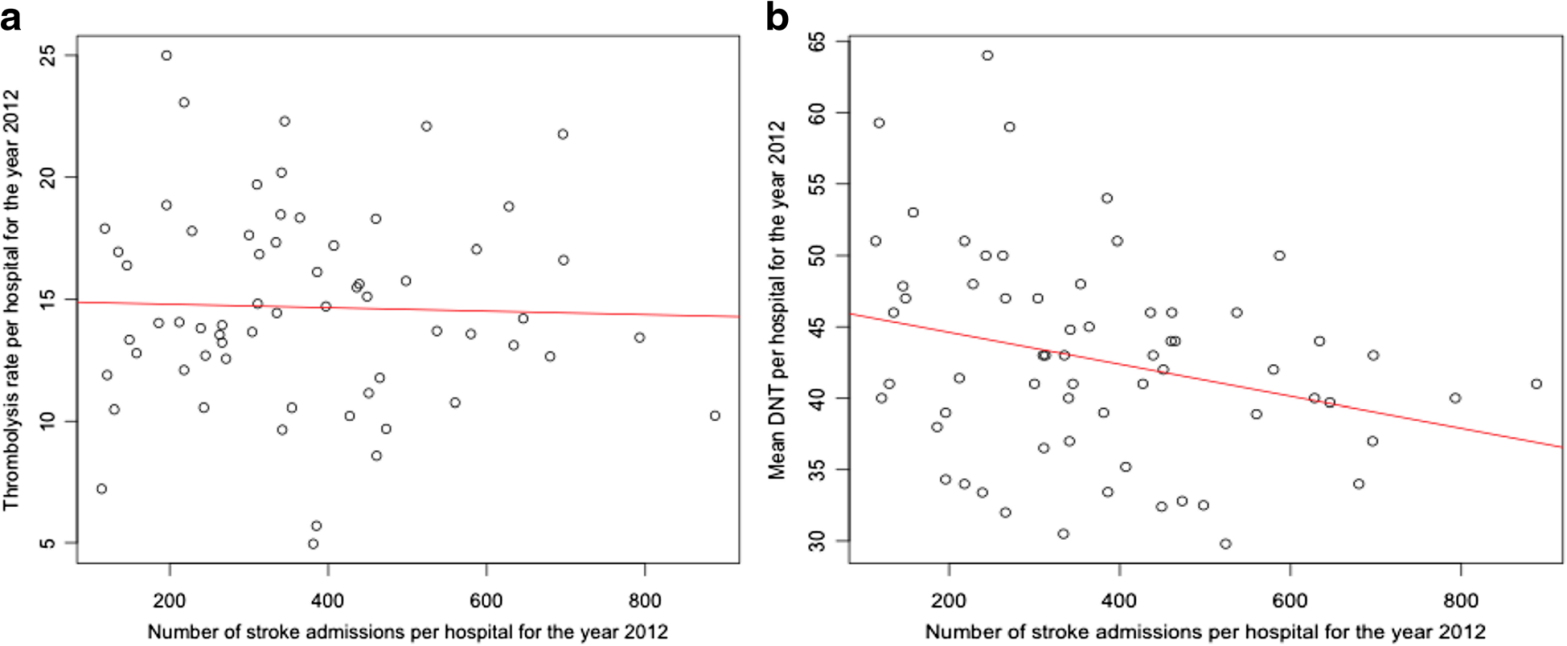
Overview of thrombolysis rates and door-to-needle time per hospital in 2012. Scatterplots showing the thrombolysis rate and door-to-needle time (DNT) by number of stroke admissions per hospital in 2012. Each dot represents the mean thrombolysis rate (**a**) or mean DNT (**b**) per hospital. The line represents the linear regression line with *R* ^2^ = 0.001 and *p* = 0.808 (**a**) and *R* ^2^ = 0.08 and *p* = 0.027 (**b**)

We found no correlation between higher stroke volume and higher thrombolysis rates (Fig. 2a: *R*^2^ = 0.001, *p* = 0.808), and a weak but significant correlation suggesting that higher stroke volume centres had lower DNT (Fig. 2b: *R*^2^ = 0.08, *p* = 0.027). We found no correlation between mean age (*r* = −.037 and *r* = 0.019) or gender (*r* = 0.158 and *r* = −.008) and mean thrombolysis rate or mean DNT respectively. Mean thrombolysis rates higher in academic hospitals (16.7 %) compared to non-academic referral centers (15.4 %) and community hospitals (14.0 %) but the difference was not significant (*p* = 0.251). Mean DNT was 39.6 min for academic hospitals, 39.3 min in non-academic referral centers and 45.1 min in community hospitals (*p* = 0.005).

After adjusting for mean age, gender and type of hospital, total stroke volume was a not significant independent predictor for thrombolysis rate (Table 2, *p* = 0.243, Beta = −.224) nor for mean DNT (Table 3, p = 0.979, Beta = 0.005). Type of hospital was no independent predictor of thrombolysis rate or for mean DNT.

**Table 2:** Linear regression analysis of possible independent predictors for mean thrombolysis rate per hospital

		Beta	*p*
Age		0,182	0,172
Gender		0,109	0,424
Type of hospital			
	Academic (reference)	0	0
	Non-academic referral	0,409	0,418
	Community	0,083	0,87
Total stroke volume		−0,224	0,243

**Table 3:** Linear regression analysis of possible independent predictors for mean door-to-needle time per hospital

		Beta	*p*
Age		−0,15	0,242
Gender		0,045	0,732
Type of hospital			
	Academic (reference)	0	0
	Non-academic referral	−0,281	0,562
	Community	0,135	0,785
Total stroke volume			

## Discussion

In this study we aimed to assess the trends in national thrombolysis rates and door-to-needle times in the Netherlands between 2005–2012 and the relationships between stroke patient volume per hospital, thrombolysis rates and door-to-needle time (DNT). We found that thrombolysis rates strongly increased throughout the years while DNT decreased. Hospital volume was no independent predictor of thrombolysis rate nor of DNT.

### Trends in thrombolysis rates and DNT

We observed an increase of national IVT rates from 6.4 % in 2005 to 14.6 % in 2012 in Dutch hospitals. This increase corresponds to gradually increasing thrombolysis rates in other western countries and regions [5–9]. In many countries campaigns have tried to improve quality of stroke care including thrombolysis rates by adapting guidelines for acute stroke care, improve logistic processes within hospitals, organization of regional referral systems, telestroke and improvement of the public awareness for stroke [22–24]. The increased thrombolysis rates suggest that these measures altogether were effective. That the routines within hospitals similarly have improved, is reflected in decreased DNT, from 72.7 min in 2005 to 41.4 min in 2012. None of the trends were caused by the entrance of new hospitals over the years. A sensitivity analysis with exclusion of the seven hospitals entering the registry in 2011 led to the same difference in results, as well as excluding a subgroup of all 31 hospitals participating both in 2008 and 2012.

There was a stabilisation of IVT utilization between 2010 and 2012. Patient characteristics (mean age, gender, stroke type) remained stable over all of the years and as the trend in decreasing DNT flattened as well, it is possible that the current thromobolysis rates and DNT reflect the maximum possible on a national scale, without further interventions. However, thrombolysis rates are not optimal [3] and may increase. It is likely that further improvements should be sought in fighting pre-hospital delays, i.e., awareness in the general public and emergency services.

### Role of volume of stroke admissions

Total volume of stroke admissions per stroke service was not an independent predictor of mean thrombolysis rate nor of mean DNT. For the year of 2012 we also found no significant relationship between volume of stroke admissions and mean thrombolysis rates or mean DNT, when corrected for age, gender and type of hospital. There was a trend, towards higher thrombolysis rates in larger centers. When looking at the relationship between volume and DNT there seems to be a weak but significant relationship. Significance disappears when correction is used for age, gender and type of hospital. Interestingly, academic centers had higher thrombolysis rates, and lower DNTs together with non-academic referral centers, as compared to regional hospitals. So, even though there is a trend suggesting that larger volumes account for better results, this by no means reaches statistical significance, but organisational issues play a role in these process measures. This finding is in line with many previous studies showing no relationship between volume and processes or outcomes [16, 17, 25]. In contrast, there have been studies that demonstrated a positive relationship [26–28]. In general, these studies still lack to identify underlying reasons for higher thrombolysis rates and faster DNT in high volume hospitals. Other factors that might influence “in hospital” delay include transport between the emergency department to the CT scan laboratory or stroke department [29]. One study found high volume hospitals to have fewer delays between arrival and brain scanning [28]. Waiting for laboratory results, especially in patient using anticoagulants, has been reported as a delaying factor [30]. Physicians are more cautious in using thrombolytic therapy in elderly patients [25] patients with mild symptoms, or patients suffering from posterior circulation stroke [30]. Several international studies suggest that thrombolysis in early arriving patients is due to be delayed by the thought that there is enough sufficient time before the time window ends [25, 30]. The found differences between studies from different western countries is most likely due to different health care systems [25, 28].

Most studies on the influence of stroke admission volume on outcomes of stroke care so far used threshold values in their statistical analysis [13–17]. Generally, equal groups of high, medium and low hospitals are created. The cut-off values that are implied by this method have a direct influence on the conclusions [16, 18, 19]. Besides this statistical flaw in using thresholds, they are clinically implausible as no sudden large improvement is expected when a hospital treats one patient more. There is no indication that one optimal threshold actually exists [19]. To reliably assess the relationship between volume and outcome, we chose to analyse volume on a continuous scale.

### Strengths and limitations

Our study has several strengths. First of all we used information on a large number of admissions, covering 65 hospitals in the year 2012, a majority of all stroke services in the Netherlands. This study was done nationwide and included academic, large non-academic and community hospitals. Most studies on the effect of patient volume in stroke care so far focused exclusively on mortality rate as an outcome, without looking at potential mediating factors between volume and outcome. In this study we aimed at the relationship between stroke patient volume, thrombolysis rates and DNT, thereby looking at possible underlying mechanisms through which higher volume may cause better outcomes.

There are some potential limitations to our analysis. Our data result from a yearly voluntary survey and participating stroke services are self-selected. Participating hospitals may have more focus on quality improvement in stroke care than non-participating centers, limiting the generalizability of our results. Mean thrombolysis rates and DNT of non-participating hospitals may have improved less than our national averages. For the first three years information was collected on stroke services level. In some areas a stroke service comprised multiple hospitals making it difficult to compare hospital level information between 2005–2007 and the years after. Only information on hospital level was available, so we were not able to perform corrections for individual patient characteristics. This may have introduced bias when associations at the group level do not represent associations at the individual level.

Additionally, we did not have information on stroke severity, which is an important case-mix factor in stroke. Limited data on post-admission functional status with Barthel index (not presented here) do not point to large differences in stroke severity. Data on patient outcome (e.g., mortality) were insufficiently available in this study and therefore could not be used. A final limitation is that we present data on hospital admissions, it is possible that one patient had more than one admission in the same year.

### Implications

In view of a current debate whether norms merely based on patient numbers should be used for selecting stroke centers, we found no clear relationship between volume and intravenous thrombolysis rate and DNT. The current literature does not provide evidence for a volume threshold-based policy. Future extension of the current stroke registration to individual patient data will help us to further examine underlying processes in stroke care that potentially mediate a relationship between volume and outcome. It should be emphasized that our conclusion only concerns the relationship between volume and outcome in intravenous thrombolysis. In view of the proven effectiveness of mechanical thrombectomy, and hence the increasing use of this treatment, the use of this treatment will increasingly determine the quality of stroke care. Because mechanical thrombectomy is technically a more complicated procedure it is possible that in this treatment volume actually does have an influence on the ultimate outcome. A better understanding of the mechanisms explaining the debated positive relationship between volume and outcome in stroke might help us to improve acute stroke care more than use of single threshold values can.

## Conclusion

Intravenous thrombolysis rates in the Netherlands more than doubled between 2005 and 2012 from 6.4 % to 14.6 %, and there was a large decline in mean DNT. When corrected for age, gender and type of hospital, we found no evidence for a relationship between stroke admission volume per hospital and mean thrombolysis rate and door-to-needle-time. Thus, our data do not support a solely volume based policy of selection of stroke centres for thrombolytic therapy. Yet organisational issues are relevant. Future extension of our registration to individual patient data will help further improvement of stroke care.
